# CHARACTERIZING PERFORMANCE VARIABILITY ACROSS COGNITIVE TASKS IN OLDER ADULTS WITH AND WITHOUT ADHD

**DOI:** 10.1093/geroni/igac059.2956

**Published:** 2022-12-20

**Authors:** Laura M Klepacz, Eric Cerino, Almar A L Kok

**Affiliations:** Northern Arizona University, Flagstaff, Arizona, United States; Northern Arizona University, Flagstaff, Arizona, United States; Amsterdam University Medical Center, Amsterdam, Noord-Holland, Netherlands

## Abstract

Most research on cognitive performance among individuals with Attention Deficit/Hyperactivity Disorder (ADHD) focuses on younger persons and on cognitive variability within speeded response-time tasks. Dispersion, i.e., variability across a range of cognitive domains, is emerging as a promising indicator of age-related and pathological cognitive impairment. However, there has yet to be an evaluation of differences in dispersion among older adults with and without ADHD. We address this gap by assessing associations of age and ADHD status with dispersion. We hypothesize older adults will exhibit greater dispersion than comparatively younger adults and explore whether individuals with ADHD exhibit greater dispersion than individuals without ADHD. In a sample of 231 adults from the Longitudinal Aging Study Amsterdam (Average age=71.64 years, SD=7.7, 59% female), 23 individuals met DSM-IV criteria for ADHD and 208 were classified as neurotypically functioning. Participants completed 13 tasks spanning domains of attention, fluency, memory, processing speed, and reasoning. Dispersion across the tasks was calculated as an intraindividual standard deviation. We regressed dispersion on age and ADHD status and adjusted for sex. Older age was significantly associated with greater dispersion (Est =0.06, SE=0.03, p=0.01). However, dispersion profiles did not vary as a function of ADHD status (Est.=-0.25, SE=0.67, p>05). Preliminary results suggest that dispersion across cognitive tasks may not be a sensitive marker of ADHD in older adults, although statistical power to detect differences was relatively low in the current study. As expected, age was a significant predictor of increased dispersion, consistent with accounts of age-related changes in neurological integrity.

